# Photoactivity and optical applications of organic materials containing selenium and tellurium

**DOI:** 10.1039/c9sc04279b

**Published:** 2019-10-07

**Authors:** Gabrielle C. Hoover, Dwight S. Seferos

**Affiliations:** a Department of Chemistry , University of Toronto , 80 St. George Street , ON M5S 3H6 , Canada . Email: dwight.seferos@utoronto.ca; b Department of Chemical Engineering and Applied Chemistry , University of Toronto , 200 College Street , Ontario M5S 3E5 , Canada

## Abstract

Sulfur-containing compounds, particularly derivatives of thiophene, are well studied for organic optoelectronic applications. Incorporating selenium or tellurium in place of sulfur imparts different physical properties due to the fundamental differences of these atoms relative to their lighter analogues. This has a profound influence on the properties of molecules and materials that incorporate chalcogens that may ultimately lead to new opportunities and applications. This mini-review will focus on the quantitative and qualitative photophysical characteristics of organic materials containing selenium and tellurium as well as their emerging applications as molecular photoactive species, including light-emitting sensors, triplet sensitizers, and beyond.

## Abstract 2

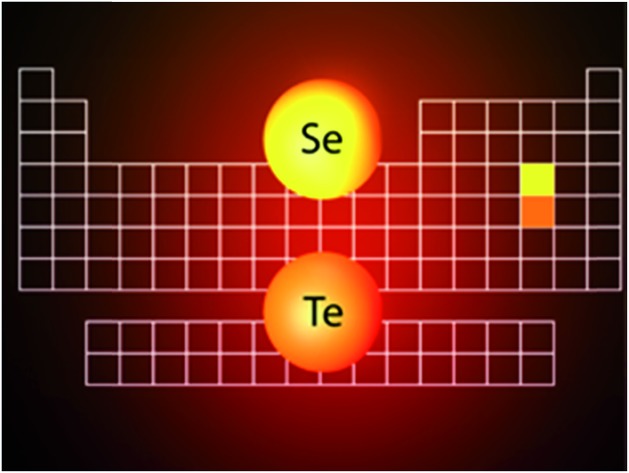
Incorporating selenium or tellurium into photoactive species imparts new photophysical properties that may be exploited in materials applications.

## 1. Introduction

The chalcogens (O, S, Se, and Te) have attracted considerable attention for materials applications in the last few decades. In the polymer electronics communities substantial attention has focused on the synthesis and properties of polythiophenes and their derivatives.^1^–^5^ In the inorganic materials space, much work has focused on semiconductor films or nanocrystals such as CdSe or PbSe quantum dots or CdTe.^6^–^12^ In between lie classes of organochalcogens, where selenium and tellurium have been judiciously placed into otherwise organic materials. Such materials have been used as photovoltaics,^13^ transistors,^14^ thermoelectric generators,^15^ light emitting devices,^16^ and sensors.^17^ The heavier chalcogens impart different physical properties than analogous lighter compounds due to fundamental differences such as atom size and bond length, electronegativity, and polarizability.^18^ These differences have a profound influence on the morphology, crystallinity, and charge transport of polymers.^19^–^21^ Many studies focus on substituting heavier chalcogens in both semiconducting polymers and small molecules, including organic photovoltaics^13^ and as n-type materials.^22^

**Fig. 1:**
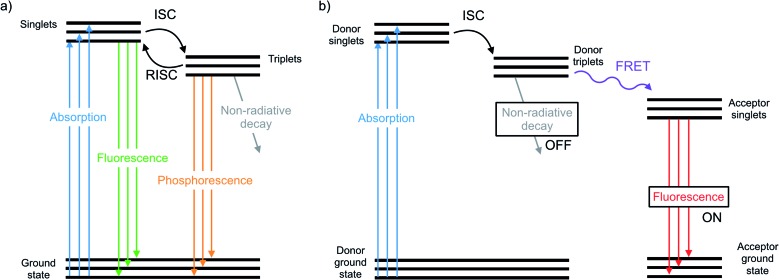
(a) Representative photophysical pathways of an organochalcogen chromophore/fluorophore include absorption, fluorescence, intersystem crossing (ISC), reverse ISC, phosphorescence, and non-radiative decay. (b) Representative photophysical pathways of an organochalcogen fluorescent probe that is off when non-radiative decay occurs through triplet quenching, and on when Förster Resonance Energy Transfer (FRET) occurs to a fluorescent acceptor.

The photophysical properties and optical applications heavier organochalcogens is also an emerging field, yet it has received somewhat less attention. This is in part because heavier organochalcogens are not traditional luminescent materials: the fluorescence that occurs in lighter chalcogens is often quenched upon substituting heavier elements. Improved spin orbit coupling resulting from the heavy atom effect promotes intersystem crossing (ISC) to rapidly populate the triplet state.^23^ The triplets will decay according one of three pathways (Fig. 1). First, phosphoresce may occur, which is uncommon at room temperature due to the spin-forbidden nature of this relaxation. Alternatively, the triplet may decay non-radiatively, which is often faster. For example, fluorescence quenching is observed when sulfur is substituted for oxygen in perylene diimides through a ^1^(n, π*)–^3^(π, π*) (S_1_–T_1_) transition.^24^ Third, the excited molecule may interact with another species nearby. These competing pathways may be modulated by chemical design and used for excited state chemistry, especially if the photoexcited molecule is close in proximity to another species. This minireview will summarize the main photophysical effects of heavy chalcogen substitution, recent advances in the understanding of how to exploit the unique photophysical properties of ‘heavy’ organochalcogens as sensors and triplet sensitizers, and will finally discuss future strategies for the further advancement of optical materials using this chemistry.

## 2. Quantitative studies on heavy chalcogen substitution effects

**Fig. 2:**
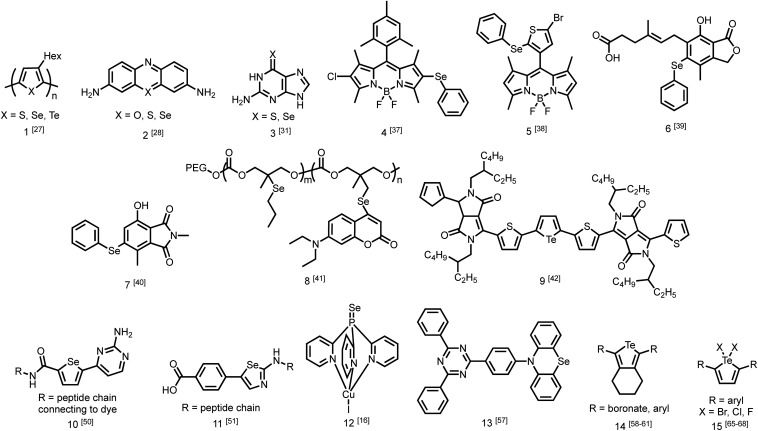
Discussed photoactive compounds containing selenium and tellurium.

While the number of studies utilizing heavy chalcogen-containing organic materials for specific applications is growing (Fig. 2),^25^,^26^ only a small number have reported the direct effects of substituting sulfur with selenium or tellurium on the rates of photophysical processes in a quantitative fashion. These studies are becoming increasingly necessary for the rational design of materials with heavy atoms, and to better understand the performance of these materials in sensors, triplet sensitizers, and other optical devices.

One study from Pensack *et al.* directly compared the photophysics of chalcogen-containing polymers with substitutions from sulfur to selenium to tellurium.^27^ The decrease in polymer photoluminescent quantum yield (PLQY) when moving down Group 16 is indicative of more rapid ISC with heavier chalcogens. The study compares three polymers (**1**) with identical solubilizing side chains: poly(3-hexylthiophene) (P3HT), poly(3-hexylselenophene) (P3HS), and poly(3-hexyltellurophene) (P3HTe). The PLQY of P3HT is 0.30, while those of P3HS and P3HTe are drastically reduced to 4.2 × 10^–3^ and 1.4 × 10^–4^, respectively. Furthermore, using transient absorption spectroscopy, this study reported simulated emission decay time constants of 600 ps for P3HT, 26 ps for P3HS, and 1.8 ps for P3HTe, suggesting rapid population of the triplet state. This study demonstrates that incorporating a heavier chalcogen results in faster ISC in organochalcogen polymers.^27^ The authors indicate that this would be advantageous for exploiting triplet excitons in future work on organic photovolatics.

Another study by Rodriguez-Serrano *et al.* directly compared the rate of ISC in oxonine, thionine, and selenine small molecule dyes (**2**) using both experimental observations and quantum mechanics.^28^ The ISC rate of oxonine had previously been experimentally determined as 7.2 × 10^5^ s^–1^.^29^ In this study, its fluorescence rate was estimated to be considerably higher at 2.10 × 10^8^ s^–1^,^28^ rendering it highly fluorescent in water with an experimental quantum yield of 1.0.^30^ By comparison, the ISC and fluorescence rates of thionine were 1 × 10^9^ s^–1^ and 1.66 × 10^8^ s^–1^, indicating that triplet state population was an order of magnitude faster than fluorescence. The population of triplet states is also supported by a singlet oxygen quantum yield of 0.53 in water. Using density functional theory, the rate of ISC in selenine was estimated to be around 1 × 10^10^ s^–1^ while the rate of fluorescence was estimated to be 1.6 × 10^8^ s^–1^. This report demonstrates that in changing from sulfur to selenium, the rate of triplet formation may improve on the scale of an order of magnitude.^28^

More recently, Farrell *et al.* demonstrated that the effect of changing from sulfur to selenium can be considerably more extreme.^31^ Transient absorption spectroscopy was used to determine the rate of ISC. Upon excitation, both thioguanine and selenoguanine (**3**) underwent rapid ISC on sub-picosecond time scales, with selenoguanine triplet population being about three times faster than that of thioguanine. Due to the heavy atom effect of selenium, however, selenoguanine relaxed from the triplet state with a time constant of 1.7 ns, more than 830 times faster than thioguanine with a time constant of 1420 ns. In this case, it was hypothesized that the yield of singlet oxygen generation would decrease due to triplet decay outcompeting the diffusion of oxygen.^31^

These studies demonstrate that it is important to increase the rate of triplet population when considering incorporation of selenium and tellurium for various applications, as faster triplet population is advantageous for materials that harvest triplet excitons. In addition, it has been highlighted that the rate of triplet decay must also be considered, as it is not desirable for non-radiative emission to outcompete other photophysical processes.

## 3. Organochalcogens as fluorescent sensors

**Fig. 3:**
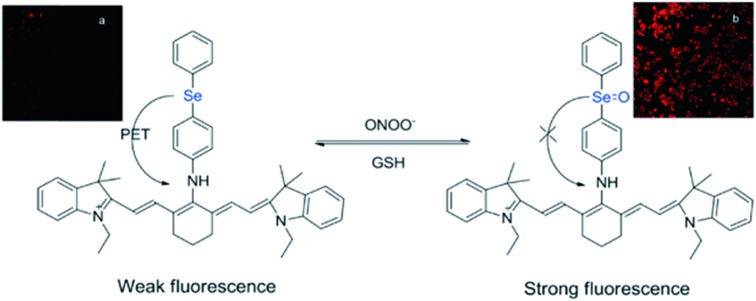
Peroxynitrite oxidation of selenium inhibits photoinduced electron transfer (PET) to a cyanine dye, resulting in fluorescence. The sensor is regenerated by exposure to glutathione. Reprinted with permission from ref. 33. Copyright 2011 ACS.

While many organochalcogens undergo population of the triplet state, others have been reported to display suppressed intersystem crossing and instead emit from the singlet state. This emission may be tailored to turn on or off upon proximity to another analyte which can quench fluorescence. This is often controlled through a photoinduced electron transfer (PET) rather than through conversion to a triplet state. In PET, upon excitation an electron is transferred from the donor to the acceptor, quenching fluorescence (Fig. 3).^32^ Through PET, organochalcogens are capable of ‘detecting’ reactive oxygen species (ROS) as well as reactive nitrogen^33^ and sulfur species.^26^,^34^ This section will briefly highlight recent developments focusing on ROS detection by organochalcogens.

The presence of ROS in cells is undesirable and indicates stress,^35^ therefore detecting ROS levels in living systems is vital to monitoring homeostasis. Selenium and tellurium are more easily oxidized than sulfur, allowing heavy organochalcogens to function as ROS sensors as their photophysical properties change upon oxidation. The Churchill group has investigated the detection of hypochlorous acid, a ROS over-produced in individuals with neurodegenerative diseases.^36^ In an initial study, the substitution of a BODIPY dye with phenyl selenide (**4**) quenches fluorescence due to photoinduced electron transfer, which was detected using spectrofluorimetry. Additional experiments showed that when the selenium is oxidized by ^–^OCl, PET is inhibited, allowing the dye to fluoresce again. However due to the insolubility of the selenide-modified BODIPY in water, it also displays aggregation induced emission without having been chemically oxidized.^37^ In a follow-up study, the authors observed π–π stacking between the selenoxide and BODIPY dye (**5**) by X-ray diffraction, which greatly enhances fluorescence.^38^ They subsequently synthesized a water-soluble organoselenide (**6**) which selectively demonstrates turn-on fluorescence in the presence of ^–^OCl.^39^ In their most recent study, they improved the stability of this acid sensor by exploring phthalates (**7**), resulting in a probe with eleven times the brightness upon oxidation of selenium.^40^

The photoluminescence change of certain organochalcogens upon exposure to ROS may be used as a turn-on sensor to indicate the release of chemicals. The Li group synthesized an organoselenide coupled to a polycarbonate (**8**) to monitor the release of an anti-tumor agent. ROS levels are elevated in cancer cells. The oxidation of the selenide was used to dissociate drug-carrying capsules in triple-negative breast cancer cells, with ROS-induced fluorescence serving as an indicator that the drug had been released.^41^ Similar sensors have also been reported using tellurium as the chalcogen. Kaur *et al.* showed that a diketopyrrolopyrrole-substituted tellurophene (**9**) undergoes PET in the unoxidized state, then upon oxidation of the tellurium, the dye fluoresces selectively in the presence of H_2_O_2_ due to inhibition of PET. This study also reported that the opposite process may be used to indicate the presence of glutathione as the fluorescence is quenched upon its reduction of tellurium.^42^

These studies demonstrate that organochalcogens with low fluorescence can be attached to dyes, allowing them to serve as sensitive and selective turn-on sensors of biological analytes such as ROS. Due to the proximity of the dye to the chalcogen, photoluminescence is prevented. An electron transfer in the excited state blocks the dye from fluorescing until the chalcogen is chemically oxidized by the analyte and PET is inhibited.

## 4. Organochalcogens as triplet sensitizers

Once organochalcogens undergo ISC into the triplet state, the triplets may be harvested rather than allowed to phosphoresce or decay non-radiatively. The triplet can be transferred to another molecule either to begin a reaction cascade (often to create singlet oxygen for photodynamic therapy)^43^ or to amplify or delay a fluorescent signal.^44^ Substitution of the chalcogen with heavier analogues red-shifts the absorption properties,^45^ allowing deeper tissue penetration because lower energy light is less prone to absorption and scattering by biomolecules.^46^ For example, thioguanine (**3**) produces singlet oxygen in 21% yield, which is useful in photodynamic therapy.^47^ Substituting selenium for sulfur shifts the absorption features of thioguanine from 341 nm and 204 nm to 357 and 209 nm, leading to an estimated 90% increase in treatment depth.^31^ While the triplet states are populated faster with the selenium analogue, they also decay 835 times faster (1.7 ns *versus* 1420 ns), resulting in an expected decrease in singlet oxygen yield due to triplet decay being much faster than oxygen diffusion. However, the triplet state may react with a nearby nucleobase more easily through π–π stacking, which would improve photosensitized damage to DNA. DFT has been used to understand that the photophysical mechanism resulting in this pronounced increase in excited state decay involves transitions between five electronic states.^48^

Triplet sensitizers can additionally help to address signal convolution, which is a large issue in fluorescent sensors for biological applications. Many fluorescent tags used to indicate the presence of biological analytes are short-lived and their emission overlaps with those of fluorescent biomolecules. Longer-lived phosphorescent tags would be advantageous because their emission would outlast that of biological autofluorescence, reducing signal convolution. However, it is challenging to design phosphorescent tags that are organic, water soluble, and luminescent at room temperature. Instead, the Uri and Enkvist groups have synthesized improved fluorescent tags by covalently linking them to triplet sensitizers. Energy from the triplet sensitizer upon excitation can be transferred to the fluorescent dyes through Förster Resonance Energy Transfer (FRET), increasing the fluorescence decay time and allowing it to persist at time scales beyond the nanosecond fluorescence of biomolecules.^44^,^49^ Without the presence of the dye, the sulfur and selenium-containing triplet sensitizers are quenched by oxygen, which occurs much faster than micro-second scale FRET to the fluorescent dye. Once the sensitizer is bound to the pocket of the protein kinase, PIM, excited triplets are sterically protected from diffusional quenching by oxygen, allowing the triplet states to persist and undergo FRET to the fluorescent dye (**10**) (Fig. 1b).^50^ The same group used the fluorescent protein, TagRFP, linked to the PIM2 protein kinase analyte rather than the triplet sensitizer (**11**) and found that FRET was more efficient over a shorter distance, with the covalent linkage resulting in a smaller transfer distance.^51^ In another study, Ligi *et al.* confirmed that while the emission spectrum of the complex is dominated by the emission of the dye, the scale of its time-gated fluorescence is determined by the persistence of the triplet state of the sensitizer.^52^ Moreover, while the sensitizer containing sulfur has a longer triplet decay time than the selenium-substituted analogue, the probes containing selenium are brighter because they undergo ISC more efficiently, leading to a higher triplet population.^53^ The authors expanded their work to a different protein kinase, CK2,^54^ and another fluorescent dye,^44^ using the same underlying method. In this series of studies, the authors have successfully utilized heavy-chalcogen substitution to maintain fluorescence using FRET, while additionally inducing a time delay suitable for avoiding signal overlap with bioluminescence when detecting biological analytes.

## 5. Future directions

Much of the work taking advantage of the photophysical properties of heavy organochalcogens has been directed toward biological applications. The improved spin–orbit coupling resulting from selenium or tellurium substitution may also improve the performance of lighting elements or displays such as organic light emitting diodes (OLEDs) when organochalcogens are used in the emitting layer. The population of triplet states and their subsequent photoluminescence could contribute to higher efficiency phosphorescent OLEDs because fewer electrons are lost to fluorescence or non-radiative decay.^55^ Alternatively, selenium substitution promotes the population of low energy triplet states, which may undergo fusion or triplet–triplet annihilation to improve the efficiency of fluorescence.^56^ As heavier chalcogens improve ISC rates, the heavy atom effect is also expected to improve reverse ISC rates to increase the efficiency of thermally activated delayed fluorescence (TADF) materials. For example, selenium conjugation to a phosphine in a Cu(i) TADF complex (**12**) resulted in dual emission from both TADF and phosphorescence.^16^ Most recently, selenium was substituted for sulfur in the donor segment of an organic donor–acceptor TADF compound (**13**), also resulting in dual emission.^57^ The effects of including heavier chalcogens in OLEDs are not yet well understood due to relatively few device-centered studies, however synthesizing organochalcogens that undergo dual emission does show great promise for accessing a library of compounds with more efficient and faster emission.

Tellurium substitution is expected to affect the photophysics of organochalcogens to a greater extent than selenium substitution. Due to an increase in the heavy atom effect, tellurium incorporation leads to more efficient and faster intersystem crossing to the triplet state compared to lighter organochalcogens.^27^ Few studies investigating the photophysical properties of organotellurides have been conducted, however, due to challenges associated with synthesizing these compounds. For example, the Churchill group attempted to study the effects of substituting tellurium for selenium in turn-on BODIPY probes, however they reported that they were unable to synthesize the desired tellurium-containing compound due to the higher bond enthalpies of tellurium.^38^ Thus, continued work on the synthesis of tellurium compounds is necessary to further understand their potential performance as photophysical materials.

Aromatic tellurium heterocycles – tellurophenes – are more synthetically accessible, and their photophysical properties have been studied by the Rivard group.^58^ It was found that tellurophenes can achieve room temperature phosphorescence when substituted with boronates (**14**).^59^ Some tellurophene derivatives displayed aggregation-induced emission,^60^ which also has potential use in materials applications. The Rivard group later synthesized phosphorescent tellurophenes with aryl group substituents at the 2- and 5-positions, greatly expanding the library of organotellurium phosphors.^55^ They have since developed additional boronate-substituted tellurophenes to be used in Suzuki–Miyaura cross coupling reactions.^61^ The continued development of organotellurium synthesis^62^ and the use of new synthetic methods when rationally designing photoactive compounds is essential for the development of new luminescent materials.

**Fig. 4:**
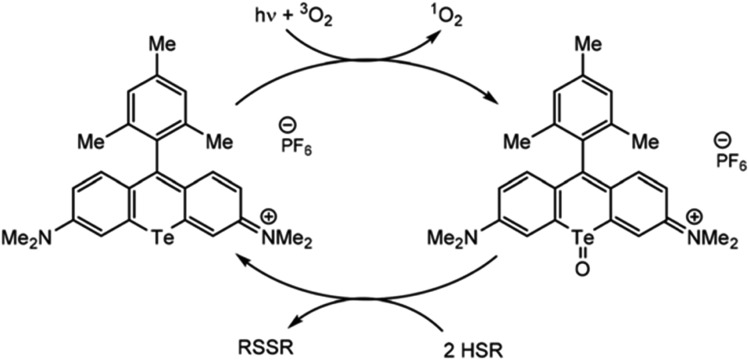
Photosensitization of singlet oxygen through the triplet state of tellurorhodamine. Reprinted with permission from ref. 64. Copyright 2019 RSC.

Just as organotellurium compounds may sensitize other compounds, they may themselves be useful photoactive reagents,^63^ potentially allowing for new types of excited state photochemistry. For example, the McCormick group demonstrated that tellurorhodamine was capable of sensitizing singlet oxygen photocatalytically to then further oxidize organo-silanes and phosphines (Fig. 4).^64^ Photoreactions of tellurophenes have additionally been studied by our group.^65^–^68^ In many of these reports the tellurium center was oxidized by halogens rather than by ROS (**15**). When the compound is photoexcited, the halogens are photoreductively eliminated.^65^ This reactivity was later expanded beyond bromine to chlorine and fluorine.^66^ The effect was then studied in tellurophenes with a variety of electron-donating and -withdrawing substituents, where electron withdrawing groups resulted in a higher quantum yield of elimination due to higher electrostatic potential.^67^ This work was expanded to include self-sensitized ROS generation, and while the tellurophenes are able to self-sensitize the generation of ROS, over-oxidation of the tellurium center leads to decomposition of the heterocycle through ring-opening.^68^ These studies suggest that photoactive species incorporating tellurium are promising to generate ROS but they must avoid decomposition to be used a photocatalysts.

## 6. Conclusions

While the optical applications of sulfur-containing organic compounds are reasonably well-understood, selenium- and tellurium-containing compounds have emerged as photoactive species, yet the photophysical mechanisms are more complex. The rapid population of triplet states and possible subsequent transfer to another molecule may amplify a signal from a fluorescent probe, transduce a signal, induce a reaction, or generate a reactive new species. The continued development of the synthesis of both selenium- and tellurium-containing materials is sure to enable even more applications beyond the examples pointed out above such as sensors and OLEDs. Future studies on the photophysics of heavy organochalcogens, including experimental determination and quantum mechanical prediction of photophysical time constants of singlet and triplet population and decay, will assist in reliably predicting their properties and will contribute to the rational design of new photophysical materials. Broadening synthetic methodologies to incorporate heavier chalcogens and gaining insight on how to manipulate photophysical time constants are both essential to unlocking the potential of selenium and tellurium in photoactive applications.

## Conflicts of interest

There are no conflicts of interest to declare.
